# Saliva as a Reliable and Non-invasive Sample for Detecting Influenza A in Severe Acute Respiratory Infection Cases

**DOI:** 10.7759/cureus.100872

**Published:** 2026-01-05

**Authors:** Junko S Takeuchi, Nobuaki Matsunaga, Ai Tsukada, Noriko Iwamoto, Noriko Fuwa, Takahiro Ichikawa, Yasuyuki Kato, Yuka Tomita, Hiroki Kitagawa, Masaya Yamato, Tetsuji Aoyagi, Hideharu Hagiya, Ryota Hase, Shuji Hatakeyama, Tohru Inaba, Koichi Izumikawa, Yoshio Takesue, Moto Kimura, Norio Ohmagari

**Affiliations:** 1 Department of Academic-Industrial Partnerships Promotion, Center for Clinical Sciences, Japan Institute for Health Security, Tokyo, JPN; 2 Antimicrobial Resistance (AMR) Clinical Reference Center, Japan Institute for Health Security, Tokyo, JPN; 3 Disease Control and Prevention Center, Japan Institute for Health Security, Tokyo, JPN; 4 Department of Infectious Diseases, Sapporo City General Hospital, Hokkaido, JPN; 5 Department of Infectious Diseases, International University of Health and Welfare (IUHW) Narita Hospital, Chiba, JPN; 6 Department of Infectious Diseases, Japanese Red Cross Aichi Medical Center Nagoya Daini Hospital, Aichi, JPN; 7 Department of Infectious Diseases, Hiroshima University Hospital, Hiroshima, JPN; 8 Department of General Internal Medicine and Infectious Diseases, Rinku General Medical Center, Osaka, JPN; 9 Department of Clinical Infectious Diseases, Tohoku University Graduate School of Medicine, Miyagi, JPN; 10 Department of Infectious Diseases, Okayama University Graduate School of Medicine, Dentistry and Pharmaceutical Sciences, Okayama, JPN; 11 Department of Infectious Diseases, Japanese Red Cross Narita Hospital, Chiba, JPN; 12 Division of Infectious Diseases, Jichi Medical University Hospital, Tochigi, JPN; 13 Department of Infection Control and Laboratory Medicine, Kyoto Prefectural University of Medicine, Kyoto, JPN; 14 Department of Infectious Diseases, Nagasaki University Graduate School of Biomedical Sciences, Nagasaki, JPN; 15 Department of Infectious Diseases, Chita Peninsula General Medical Organization, Chita Peninsula Rinku Hospital, Aichi, JPN

**Keywords:** influenza a, nasal vestibular swab, nasopharyngeal swab, rapid diagnostics, rt-qpcr, saliva, sari

## Abstract

Background

Nasopharyngeal swab sampling remains the gold standard for influenza diagnosis; however, it has several limitations, including dependence on medical staff, invasiveness, potential for nosocomial transmission, and occupational exposure risk. Non-invasive alternatives, such as saliva and nasal vestibular swabs, may improve patient comfort and participation in clinical studies. In addition, diagnosis with reverse transcription real-time quantitative polymerase chain reaction (RT-qPCR) is often delayed because it requires trained laboratory technicians and facilities with appropriate laboratory settings. Although rapid diagnostic devices such as the GenPad® offer potential alternatives to RT-qPCR, their performance with non-invasive samples remains insufficiently explored. This study addresses the two key questions for influenza detection in severe acute respiratory infection (SARI) cases: (i) whether saliva or nasal vestibular swab samples serve as suitable alternatives to nasopharyngeal swab samples, and (ii) whether the GenPad® provides a reliable option for detecting influenza using saliva samples.

Methodology

A prospective observational study was conducted with 16 inpatients classified as having SARIs and diagnosed with influenza between December 2024 and March 2025 in Japan. Paired saliva and nasal vestibular swab samples were collected 1-9 (median = 3.5) days after symptom onset. RT-qPCR testing was performed according to the National Institute of Infectious Diseases protocol. Saliva samples were also tested using the GenPad® system. Comparisons between sample types and diagnostic methods were analyzed using the exact McNemar's test.

Results

Among the 16 influenza-positive patients, saliva samples demonstrated higher sensitivity (87.5%) than nasal vestibular swabs (31.3%) in RT-qPCR when compared with the diagnostic results obtained from nasopharyngeal swabs. A comparison of RT-qPCR results between saliva and nasal vestibular swabs revealed a total agreement of 43.8%, with exact McNemar's test showing a significant difference (p = 0.0039). While nasal vestibular swabs showed inconsistent results, saliva samples consistently tested positive, particularly within seven days of symptom onset (100% positive agreement). The GenPad®, a rapid diagnostic device, showed promising performance (92.9%) using saliva samples compared to RT-qPCR.

Conclusions

Saliva is a reliable non-invasive alternative to nasopharyngeal swabs for influenza detection in SARI cases, particularly within seven days of symptom onset, whereas nasal vestibular swabs show lower sensitivity. Additionally, the GenPad® provides comparable performance to RT-qPCR using saliva samples, offering a rapid, portable diagnostic option. These approaches may mitigate discomfort, minimize infection risk for healthcare workers, and improve testing capacity. However, the absence of influenza-negative controls and the small sample size (n = 16) substantially limit the assessment of diagnostic accuracy and specificity. As a result, the broader applicability of our findings should be interpreted with caution, and further studies are required to validate these observations.

## Introduction

The coronavirus disease 2019 (COVID-19) pandemic has prompted a more comprehensive surveillance of acute respiratory infections (ARIs), including COVID-19, respiratory syncytial virus (RSV) infection, and influenza, worldwide [1]. Countries use surveillance systems such as the Respiratory Virus Hospitalization Surveillance Network (RESP-NET; the United States) [2], the Severe Acute Respiratory Infection Watch (SARI Watch; the United Kingdom) [3], and the European Respiratory Virus Surveillance Summary (ERVISS; European Union) [4]. In Japan, clinical information and specimens derived from severe acute respiratory infections (SARIs) have been added to the Infectious Disease Clinical Research Network With National Repository (iCROWN, the successor to REBIND) since September 2024 [5]. Furthermore, on April 7, 2025, ARI has been designated a “Category V Infectious Disease” under Japan’s Infectious Disease Control Law [6] and is subject to sentinel surveillance.

As a representative SARI/ARI, influenza requires early diagnosis to appropriately guide antiviral therapy and patient isolation for effective disease control [7,8]. Antiviral treatment is most effective when initiated within 48 hours of symptom onset, helping to reduce the likelihood of hospitalization or the need for critical care, as well as to prevent further transmission within the community. However, at the beginning of the COVID-19 pandemic, two major challenges in diagnosing severe acute respiratory syndrome coronavirus 2 (SARS-CoV-2) were highlighted. First, sample collection relied exclusively on healthcare workers. Second, reverse transcription real-time quantitative polymerase chain reaction (RT-qPCR)-based diagnosis was delayed due to the need for specialized personnel and facilities. In response, self-sampling and point-of-care testing (POCT) methods for molecular diagnosis were developed [9].

Although nasopharyngeal swab sampling remains the gold standard for influenza diagnosis, it requires medical personnel and can be uncomfortable, especially for children. Amid pandemics, staff shortages and the risk of aerosol generation during nasopharyngeal swab sampling further complicate this approach. Less invasive, self-collectable methods such as saliva or nasal vestibular (anterior nasal cavity) swab sampling offer practical alternatives. These simpler collection methods may also increase participation rates in clinical studies and surveillance programs. Owing to the high sensitivity of RT-qPCR, detection rates across different sample types in influenza virus may be comparable [10]. Multiple studies on influenza detection have reported high concordance rates (93%-100%) between saliva and nasopharyngeal samples [11-18]. However, studies comparing saliva and anterior nasal swab samples remain limited. Although two studies have reported that saliva appeared less effective than self-collected anterior nasal swabs for detecting influenza A, these studies included only a small number of influenza-positive cases (n = 8 [19] and n = 1 [20]). Therefore, the evidence remains insufficient to draw firm conclusions about differences in detection performance between saliva and anterior nasal samples.

RT-qPCR is the gold standard for influenza detection because of its high sensitivity. However, it typically takes two to three hours, including RNA extraction, and requires specialized facilities and a stable power supply, limiting its application in smaller or rural clinical setups and in urgent settings [21]. To address these challenges, the GenPad® (MIRAI GENOMICS, Kanagawa, Japan) represents a promising alternative. It utilizes isothermal nucleic acid amplification based on SmartAmp (a Smart Amplification Process) and fluorescence detection [22,23], offering a portable, battery-operated solution with results within 50 minutes. However, few studies have examined its performance using clinical samples.

Therefore, this study addresses the following two key questions in SARI diagnostics: (i) whether saliva or nasal vestibular swab samples serve as suitable alternatives to nasopharyngeal swab samples, and (ii) whether the GenPad® provides a reliable option for detecting influenza using saliva samples.

## Materials and methods

Ethics statement

This study was approved by the Ethics Review Committee of the National Center for Global Health and Medicine, Japan (NCGM-S-004948). All methods were performed in accordance with relevant guidelines and regulations.

Study participants

We recruited inpatients who met certain criteria at the time of admission in Japan. The case-cohort criteria for SARI were: (i) All of A-C: (A) Respiratory pathogens were detected or strongly suspected. (B) Airway symptoms, such as cough. (C) At the time of admission, the patient was not in a care facility, undergoing tracheotomy, or having confirmed significant dysphagia. (ii) One or more of D-F: (D) Chest imaging showed infiltrates. (E) SpO² was ≤94% under room air conditions. (F) New oxygen therapy, invasive mechanical ventilation, or non-invasive mechanical ventilation was introduced. All patients gave informed consent for participation in the prospective observation study.

In total, 16 patients classified as having SARIs and diagnosed with influenza participated in this study. Patients were diagnosed at day 0−4 (median = 1) after onset at each medical facility using one of the following methods with nasopharyngeal swab samples: film array test, rapid test kit, or antigen test (the day of onset was designated as day zero). There were 13 (81.25%) male participants aged 16-82 (median = 67.5) years. All clinical samples were collected 1−9 (median = 3.5) days after symptom onset. Paired samples (saliva and nasal vestibular samples) were collected from each patient on the same day. The nasal vestibular samples were collected by healthcare professionals using a swab and preserved in 3 mL of Universal Transport Medium (FLOQSwabs® and UTM®, 307C; Copan, Brescia, Italy). Saliva samples were self-collected by participants under the supervision of healthcare professionals and were stored in sterile tubes without preservative medium using a saliva collector (SP1-RO0003-56, AXIS Inc., Ibaraki, Japan). The participants avoided gargling, brushing their teeth, or eating within 30 minutes before sample collection. All residual samples were stored at −20 or −80°C until use.

Nucleic acid extraction

Each saliva sample (150 µL) was diluted with an equal volume of phosphate-buffered saline without calcium and magnesium (PBS(-)), vigorously mixed, and spun down. Nucleic acid (60 µL) was extracted from 200 µL of sample, either saliva (diluted two-fold with PBS(-)) or nasal vestibular swab suspended in 3 mL of UTM, using a KingFisher APEX System (Thermo Fisher Scientific, Waltham, MA, USA) and the MagMAX Prime Viral/Pathogen NA Isolation Kit (A58145; Thermo Fisher Scientific), with 60% ethanol used as the second wash buffer.

Reverse transcription real-time quantitative polymerase chain reaction

RT-qPCR was performed according to the Influenza Diagnostic Manual (5th Edition, August 2023) published by the National Institute of Infectious Diseases (NIID), Japan [24]. Briefly, RT-qPCR was performed on a QuantStudio®3 real-time PCR system (Thermo Fisher Scientific) using AgPath-IDTM One-Step RT-PCR Reagents (AM1005; Thermo Fisher Scientific) with the following primer/probe sets: the influenza A set comprised (MP-39-67For (5’-CCMAGGTCGAAACGTAYGTTCTCTCTATC-3’), MP-183-153Rev (5’-TGACAGRATYGGTCTTGTCTTTAGCCAYTCCA-3’), and MP-96-75ProbeAs (5’-FAM-ATYTCGGCTTTGAGGGGGCCTG-MGB-3’)); the influenza B set consisted of (NIID-TypeB TMPrimer-F1 (5’-GGAGCAACCAATGCCAC-3’, NIID-TypeB TMPrimer-R1 (5’-GTKTAGGCGGTCTTGACCAG-3’), and NIID-TypeB Probe2 (5’-FAM-ATAAACTTYGAAGCAGGAAT-MGB-3’)). Subsequently, 5 µL of the extracted nucleic acid was used as the template in 25 µL of an RT-qPCR reaction mixture using the following conditions: 50°C for 10 minutes, 95°C for 10 minutes, followed by 45 cycles of 95°C for 15 seconds, 56°C for 30 seconds, and 72°C for 15 seconds. The data were analyzed using QuantStudio™ Design & Analysis Software v1.5.2. The test was considered successful when amplification and no-amplification curves were confirmed in the positive and negative (nuclease-free water) controls, respectively. A sample was considered positive when the amplification curve increased. The positive RNA controls were kindly provided by the NIID (for influenza A: (A/Wisconsin/67/2022, AH1 pdm S2 +hCK1); for influenza B: (B/Austria/1359417/2021, B Victoria S1C4/ C2 +HCK1)).

GenPad system

The GenPad® Smart CoV2/FluA/FluB Research Use Only kit (RR072; MIRAI GENOMICS) was used on a GenPad® device according to the manufacturer’s protocol with minor modifications. In this study, saliva samples were collected directly into sterile tubes using a saliva collector, rather than using swabs as instructed in the kit protocol. Because the swabs included in the kit typically absorb approximately 150 µL of saliva, we used an equivalent volume of saliva to maintain consistency with the manufacturer’s protocol. Briefly, a saliva sample (150 µL) was added to a tube containing a swab suspension buffer (SSB; 2.0 mL). After inversion mixing, a nozzle cap was attached to the tube, and the extracted mixture was added to the Sample Loading Level of the GenPad Smart test cartridge. When the filter of the nozzle cap became clogged due to the viscosity of the saliva sample, it was replaced with a new cap.

Statistical analysis

Comparisons of RT-qPCR results between saliva and nasal vestibule samples, as well as between RT-qPCR and GenPad results using saliva samples, were evaluated using the exact McNemar's test (mcnemar.exact) from the exact2x2 package (v1.7.0) [25]. Positive and negative agreement rates were obtained using the epi.tests function of the epiR package (v2.0.88), with 95% confidence intervals (CIs) calculated using the Wilson method [26]. To assess differences in threshold cycle (Ct) values between saliva and nasal vestibular swabs, survival analysis was performed with undetected samples treated as right-censored at a Ct of 40. Kaplan-Meier curves were generated using the survfit function to compare the proportion of undetected results between sample types, and differences were evaluated using a stratified log-rank test with the survdiff function (stratified by case number) from the survival package (v3.8-3). The correlation and p-value between RT-qPCR Ct values and GenPad® threshold time (Tt) values were calculated using Spearman’s rank correlation. All statistical analyses were performed using R 4.3.1 (R Foundation for Statistical Computing, Vienna, Austria).

## Results

We tested paired saliva and nasal vestibular samples collected on the same day from 16 patients classified as having SARI and diagnosed with influenza between December 2024 and March 2025 in Japan. All patients, except for case number 8, had either no history of influenza vaccination or an unknown vaccination status. All samples that tested positive by RT-qPCR were identified as influenza A, and no influenza B was detected. Although the RT-qPCR in this study was qualitative and lacked a calibration curve, all samples were tested on the same plate, except for number 16, allowing Ct-based comparisons. Although number 16 was tested separately, its positive control Ct value (31.4) was equivalent to the other assay (31.6), so it was included in the analysis.

Comparison with diagnostic results

We first compared the diagnostic results obtained from nasopharyngeal swabs (collected at medical facilities using one of the following methods: film array test, rapid test kit, or antigen test) with the RT-qPCR results of the saliva and nasal vestibular samples. As this study included only cases diagnosed with influenza, a positive agreement rate could be calculated: 87.5% (14 of 16) for saliva and 31.3% (5 of 16) for nasal vestibular swab samples. Diagnosis using nasopharyngeal swabs was performed 0-4 days (median = 1) after symptom onset, which may have led to an underestimation of the sensitivity of saliva and nasal vestibular samples that were collected 1-9 days (median = 3.5) after symptom onset. This temporal discrepancy in sampling should be considered when interpreting the positive agreement rates.

Saliva versus nasal vestibular samples

**Figure 1:**
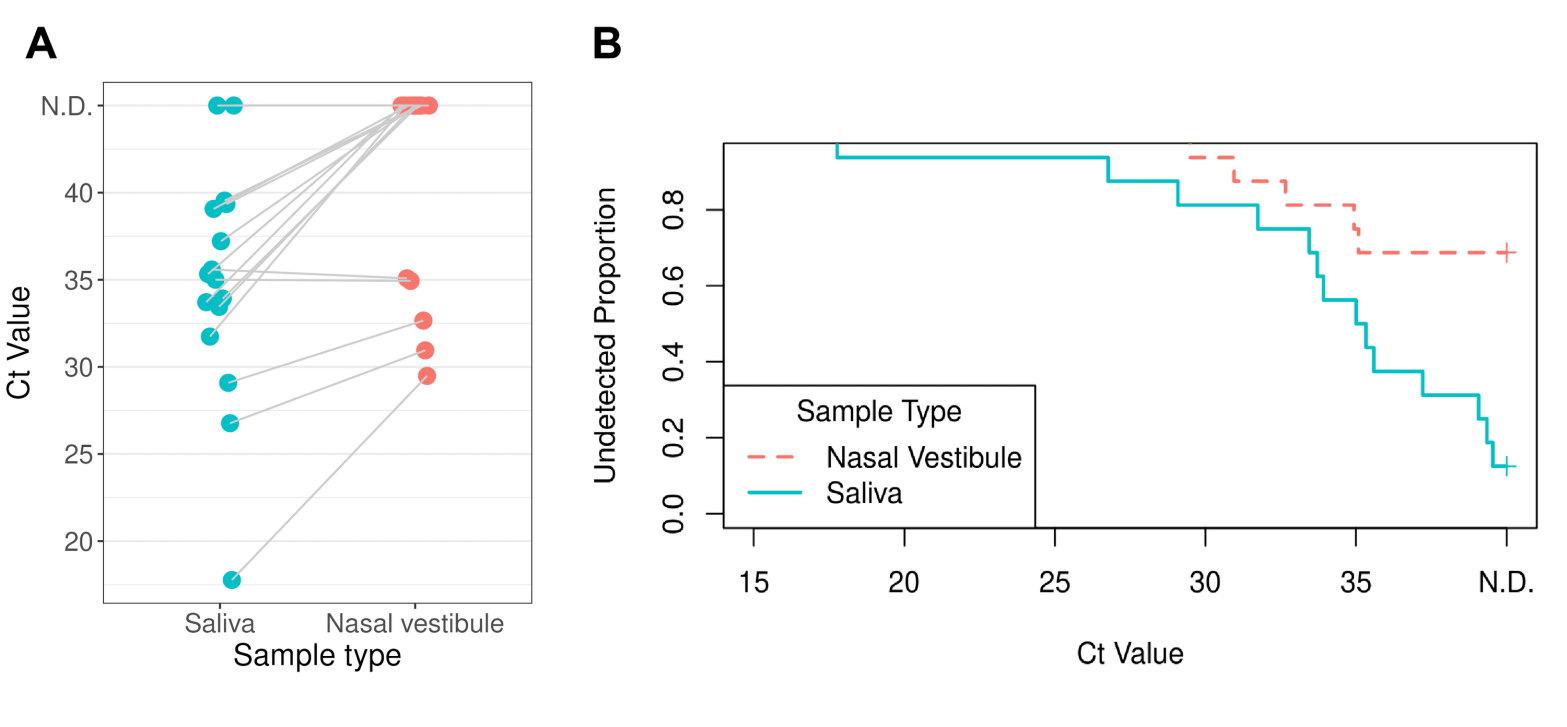
Ct values obtained by RT-qPCR among different sample types. (A) Comparison of Ct values obtained by RT-qPCR among different sample types. (B) Comparison of the proportion of undetected samples between saliva and nasal vestibule samples using Kaplan–Meier analysis. Undetected samples were treated as right-censored at a Ct of 40. Blue and pink circles/lines represent saliva and nasal vestibular samples, respectively. RT-qPCR = reverse transcription real-time quantitative polymerase chain reaction; Ct = threshold cycle; N.D. = not detected (there was no detectable amplification)

Subsequently, we compared the RT-qPCR results of the paired saliva and nasal vestibular samples (Figure 1). The positive, negative, and total agreement rates were 35.7% (95% CI = 16.3%-61.2%), 100% (34.2%-100%), and 43.8% (23.1%-66.8%), respectively, using saliva as the reference (Table 1). A significant difference in detection sensitivity between saliva and nasal vestibular samples was observed using exact McNemar's test (p = 0.0039; b = 9, c = 0; OR = ∞, 95% CI = 1.97-∞), indicating that the positivity rate was higher in saliva samples compared with nasal vestibular samples. When saliva Ct values were ≥37, those of nasal vestibular samples were undetectable (n = 6), whereas nasal vestibular samples were also detectable when saliva Ct values were ≤30 (n = 3) (Figure 1A). Furthermore, differences in Ct values between the two sample types were evaluated using survival analysis, treating undetected samples as right-censored at a Ct value of 40. Kaplan-Meier curves demonstrated that the proportion of undetected samples differed between the two sample types, and a stratified log-rank test (stratified by case number) confirmed that this difference was statistically significant (chi-square = 7.14, df = 1, p = 0.0075) (Figure 1B).

**Table 1:** Comparison of RT-qPCR using saliva and nasal vestibule samples. RT-qPCR = reverse transcription real-time quantitative polymerase chain reaction

RT-qPCR		Nasal vestibule		
		Positive	Negative	Total
Saliva	Positive	5	9	14
	Negative	0	2	2
	Total	5	11	16

Impact of antiviral treatment and sampling timing

**Figure 2:**
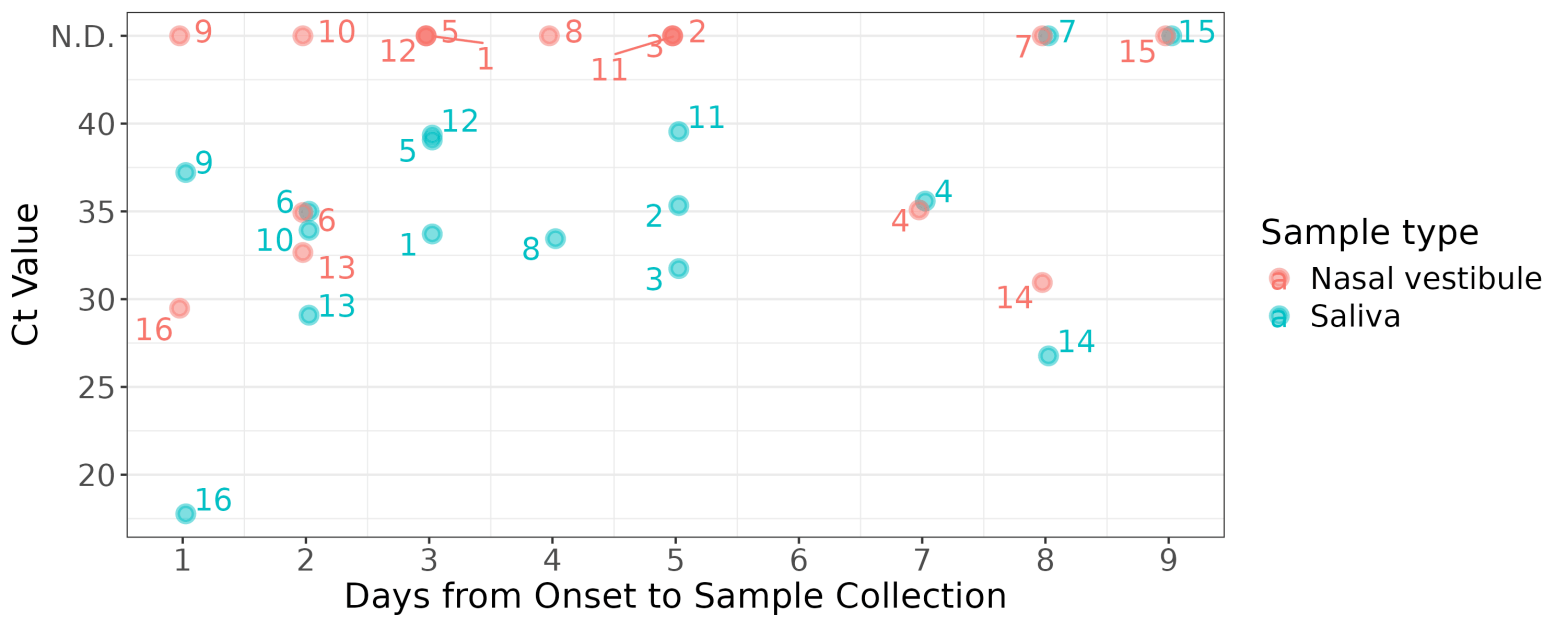
Comparison of RT-qPCR Ct values with time from symptom onset to sample collection. Days from onset to sample collection are shown on the x-axis, and Ct values are on the y-axis. Blue and pink circles represent saliva and nasal vestibular samples, respectively. RT-qPCR = reverse transcription real-time quantitative polymerase chain reaction; Ct = threshold cycle

Next, we evaluated the relationship between clinical information and the Ct values of the RT-qPCR. Before sample collection, antiviral treatment had been administered in 11 out of 16 cases: peramivir was given to numbers 1-7 and 14; oseltamivir to numbers 8, 13, and 15; and no antiviral treatment was given to numbers 9-12 and 16. Although numbers 7 and 15 (both of whom had received antiviral treatment) tested negative under RT-qPCR in both saliva and nasal vestibular swab samples, the fact that other treated patients yielded positive results suggested that antiviral treatment alone does not fully account for the variability in detection sensitivity. In contrast, the number of days from onset to sample collection appears to be more relevant: numbers 7 and 15 were collected on day eight or nine post-onset, when viral loads had likely been reduced, possibly explaining the negative results (Figure 2). Sample number 14 was collected on day eight and had the second-lowest Ct value (second-highest viral load). However, this patient was immunosuppressed due to complications following hematopoietic stem cell transplantation and was receiving multiple immunosuppressive therapies, suggesting prolonged viral persistence.

GenPad® rapid test performance

**Figure 3:**
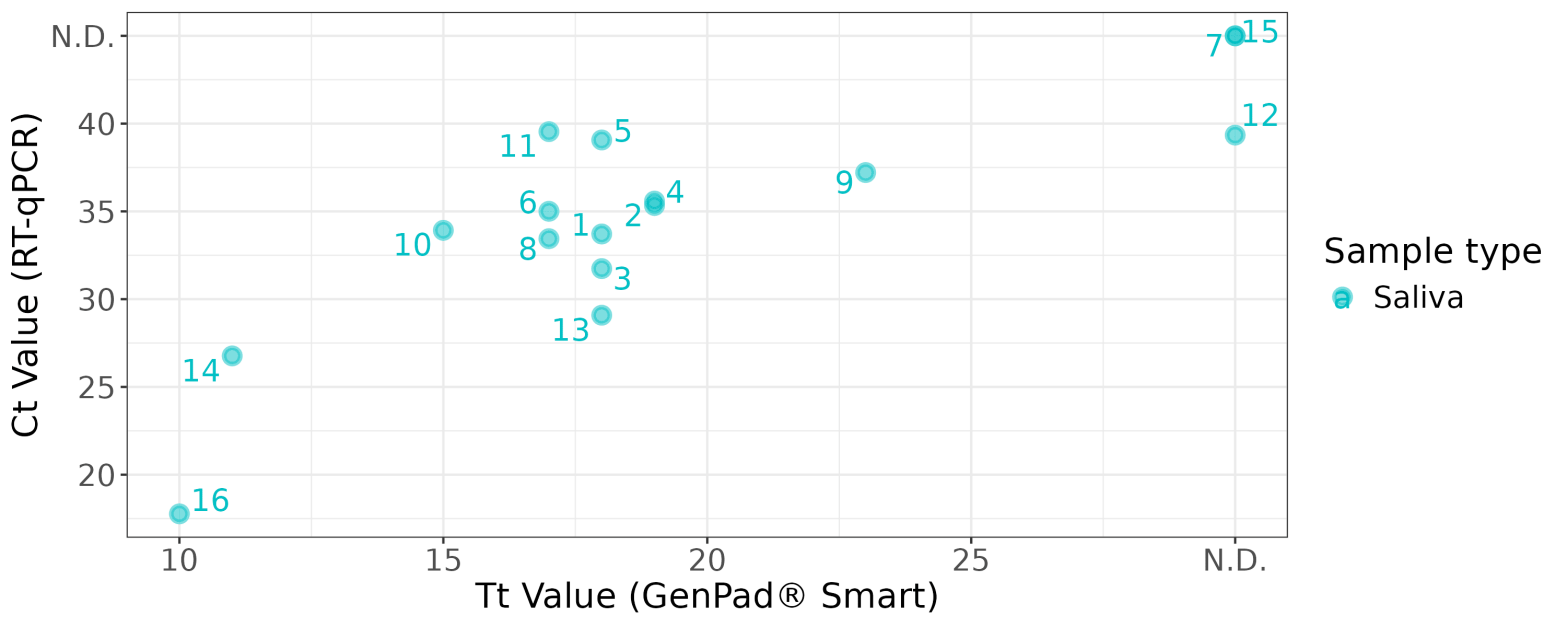
Correlation of Tt/Ct values between the GenPad and RT-qPCR methods. Blue circles indicate Tt/Ct values of saliva samples. RT-qPCR = reverse transcription real-time quantitative polymerase chain reaction; Tt = threshold time; Ct = threshold cycle; N.D. = not detected (there was no detectable amplification)

Using saliva samples, we compared the GenPad® and RT-qPCR (Figure 3). The positive, negative, and total agreement rates were 92.9% (68.5%-98.7%), 100% (34.2%-100%), and 93.8 (71.7%-98.9%), respectively, using RT-qPCR as the reference (Table 2). No significant differences in detection sensitivity between the two tests were observed using exact McNemar's test (p = 1; b = 1, c = 0; OR = ∞, 95% CI = 0.026-∞). One discordant sample, number 12, yielded a Ct value (39.5) using RT-qPCR but was not detected by the GenPad®, suggesting that the viral load was near or below the detection limit of the GenPad®. A moderate correlation was observed between Tt/Ct values of the GenPad® and RT-qPCR (Spearman’s rank correlation; rho = 0.522, S = 174.04, p = 0.067), which was not statistically significant. The GenPad® Smart CoV2/FluA/FluB kit detects Influenza A and B and SARS-CoV-2; however, only influenza A was detected in this study, consistent with RT-qPCR (although the detection of SARS-CoV-2 was not included in the RT-qPCR).

**Table 2:** Comparison of RT-qPCR and GenPad using saliva sample. RT-qPCR = reverse transcription real-time quantitative polymerase chain reaction

Saliva		GenPad		
		Positive	Negative	Total
RT-qPCR	Positive	13	1	14
	Negative	0	2	2
	Total	13	3	16

## Discussion

This study demonstrates that saliva is a more reliable non-invasive sample for influenza A detection than nasal vestibular swabs in SARI cases, particularly within seven days post-onset. Our findings differ from those of Goff et al., who demonstrated lower sensitivity of saliva compared to self-collected anterior nasal swabs [19]; however, their study included only eight influenza-positive cases, and statistical significance was not evaluated. Nevertheless, several factors may explain the apparent discrepancy between the two studies. First, our SARI cases likely included viral pneumonia (lower respiratory tract infections (LRTIs)), whereas Goff et al. [19] reported non-severe seasonal influenza cases (upper respiratory tract infections (URTIs)). These clinical differences may have involved differences in viral load distribution within the respiratory tract, potentially affecting the sensitivity of sample types. Second, our study followed strict pre-collection conditions, asking the participants to avoid gargling, brushing their teeth, or eating within 30 minutes before sample collection based on the guidelines of the Ministry of Health, Labour and Welfare. These actions interfere with viral detection in saliva samples, according to studies on SARS-CoV-2 [27,28], possibly because the impurities in saliva samples may affect PCR efficiency [29]. In contrast, in the study by Goff et al. [19] conducted before the COVID-19 pandemic, these conditions were not established for saliva collection, which may have affected virus detection. However, even when similar pre-collection conditions are applied, other factors may still influence detection sensitivity. For example, another study by Norizuki et al. [30], who tested asymptomatic travelers for SARS-CoV-2 and implemented similar pre-collection conditions for saliva sampling, reported low detection rates in saliva samples. Norizuki et al. [30] noted that the participants self-collected samples without supervision at busy airport quarantine stations, which may have affected their results. Thus, supervised saliva collection under conditions that meet pre-collection requirements may be preferable. Next, we extracted nucleic acids from the saliva samples to effectively remove impurities, ensuring that their impact on the results was minimized. In contrast, Goff et al. [19] used several rapid diagnostic kits, a condition where the substances in the saliva might have interfered with the outcomes.

The lower and variable sensitivity of nasal vestibular swabs may be related to the location of respiratory symptoms (presence of nasal discharge, URTIs vs. LRTIs), age group (children or adults), sampling techniques (rubbed vigorously or lightly, swab suspension ratio), or collection area (anterior or posterior of the nasal cavity). These results indicate that nasal vestibular swab samples may pose a risk of sampling bias. Additionally, Branche et al. reported that RT-qPCR revealed higher mean viral loads in sputum than in nasal or throat swabs for influenza A [31]. Therefore, sputum should also be considered a representative sample for influenza detection.

Most healthy volunteers ceased shedding influenza virus by day six or seven [32,33], though shedding may last longer in children or immunocompromised patients [34,35]. In our study, all saliva samples collected at ≤7 days of onset tested positive, with saliva-based RT-qPCR showing 100% positive agreement with nasopharyngeal diagnostics (Figure 2). Although pediatric cases were not included in this study, the only minor case (aged 15-19, number 4) tested positive in both saliva and nasal vestibular samples on day seven. Furthermore, although it is just one case, we confirmed that an immunosuppressed patient (number 14) can retain higher levels of the virus for an extended period, as previously reported [34].

A saliva-based rapid diagnostic kit, the GenPad®, demonstrated relatively good performance (positive agreement rate 92.9%). This aligns with a previous report showing a 100% positive concordance rate compared with that of the RT-qPCR method; however, the result was based on SARS-CoV-2 detection in saliva samples [36]. Suspension of the saliva samples in SSB containing ethanol and a chaotropic agent (guanidine thiocyanate), followed by filtration through a glass filter, may effectively contribute to the removal of impurities and elution of nucleic acids. Notably, 150 µL of saliva sample was directly treated with SSB in this study despite the kit’s protocol specifying swab-collected saliva; therefore, further evaluation is warranted. The COVID-19 pandemic highlighted the use of saliva as a sensitive and reliable diagnostic sample, especially for nucleic acid testing [18,37]. A growing body of evidence supports its use of saliva for detecting other respiratory pathogens, including influenza. This may promote the approval of saliva-based diagnostic tests for influenza. Although not assessed in this study, saliva has reportedly shown lower sensitivity in immunochromatographic rapid antigen tests [38].

The strength of this study is the direct comparison of two non-invasive sample types using RT-qPCR and access to detailed clinical data, such as the treatment history and the symptom onset date. However, this study had some limitations. First, we did not evaluate nasopharyngeal swabs simultaneously. Second, the samples were collected 1-9 days after the onset of symptoms, and antiviral therapy had been initiated in 11 out of 16 cases. These factors (a prolonged interval between onset and testing or prior treatment) may have reduced viral loads in both saliva and nasal vestibular swab samples, potentially lowering overall detection sensitivity. Third, the samples were small-scale (n = 16) and biased toward those obtained in SARI cases in Japan, preventing the generalizability of the results to a larger population or other settings. Fourth, because we did not include a control group of influenza-negative patients, we could not evaluate the negative agreement rate. Finally, while we confirmed the virus detection from saliva samples, further evaluation of viral characteristics is warranted, particularly to assess whether viable viruses can be isolated from saliva, as described in SARS-CoV-2 studies [39,40].

This article was previously posted to the medRxiv preprint server on May 21, 2025 [41].

## Conclusions

We demonstrated that the use of saliva, but not nasal vestibular swabs, is a potential non-invasive alternative to nasopharyngeal swabs for influenza A virus detection, particularly within seven days of symptom onset. Additionally, the GenPad® provides comparable results to RT-qPCR. These methods may reduce the burden on patients and healthcare settings, mitigate nosocomial infections, and increase participation rates when applied for clinical studies and surveillance purposes. However, the absence of influenza-negative control samples and the limited sample size substantially limit the assessment of diagnostic accuracy and specificity. As a result, the broader applicability of our findings should be interpreted with caution, and further studies with larger, well-controlled cohorts are required to validate these observations.
